# Time to switch from CLSI to EUCAST? A Southeast Asian perspective

**DOI:** 10.1016/j.cmi.2019.03.016

**Published:** 2019-07

**Authors:** T.P. Cusack, E.A. Ashley, C.L. Ling, T. Roberts, P. Turner, T. Wangrangsimakul, D.A.B. Dance

**Affiliations:** 1) Lao–Oxford–Mahosot Hospital Wellcome Trust Research Unit (LOMWRU), Microbiology Laboratory, Mahosot Hospital, Vientiane, Lao People's Democratic Republic; 2) National Infection Service, Public Health England, London, UK; 3) Myanmar Oxford Clinical Research Unit, Yangon, Myanmar; 4) Centre for Tropical Medicine and Global Health, Nuffield Department of Medicine, University of Oxford, Oxford, UK; 5) Shoklo Malaria Research Unit, Mahidol–Oxford Tropical Medicine Research Unit, Faculty of Tropical Medicine, Mahidol University, Mae Sot, Thailand; 6) Cambodia–Oxford Medical Research Unit, Angkor Hospital for Children, Siem Reap, Cambodia; 7) Mahidol–Oxford Tropical Medicine Research Unit, Faculty of Tropical Medicine, Mahidol University, Bangkok, Thailand; 8) Faculty of Infectious and Tropical Diseases, London School of Hygiene and Tropical Medicine, London, UK

Antimicrobial susceptibility testing (AST) of clinical isolates is essential for guiding therapy as well as for surveillance of antimicrobial resistance (AMR). The two most commonly used methodologies worldwide are those of the Clinical and Laboratory Standards Institute (CLSI) and the European Committee for Antimicrobial Susceptibility Testing (EUCAST). CLSI predominates in the United States and many regions outside Europe, where EUCAST is preferred. However, the global situation is evolving, with countries such as Australia recently switching to EUCAST [1]. The choice of AST methodology assumes increasing importance given the growing international focus on AMR, with both systems recommended in the World Health Organization's Global Antimicrobial Resistance Surveillance System (GLASS) [2]. Although collaboration between CLSI and EUCAST has occurred, fundamental differences have so far precluded them from merging or harmonizing breakpoints [3].

The Mahidol–Oxford Tropical Health Network (MORU) includes laboratories in Thailand, Laos and Cambodia that currently use CLSI disk-diffusion AST guidelines for both routine diagnostic and research purposes, but have recently been considering switching to EUCAST. Key perceived benefits of EUCAST include the stringency and transparency of EUCAST breakpoint-setting processes evident in detailed rationale documents, the lack of formal industry representation on EUCAST committees, and the freely available nature of all output in user-friendly format. Updated CLSI documents must be purchased annually, at a cost to non-members in 2018 of US$342–504 depending on the bundle chosen, although breakpoints for frequently isolated non-fastidious organisms can be freely accessed as an online-only companion to the CLSI M100 document [4]. While studies have examined the impact of replacing CLSI with EUCAST breakpoints, published reports of the practical laboratory implications of switching between the two methodologies are lacking, particularly for low- and middle-income countries. We report here the key practical differences between CLSI and EUCAST methodologies and the implications of adopting EUCAST guidelines in our laboratory network.

Clinical breakpoints from both organizations from 2018 [5], [6] and disk-diffusion AST guidelines from 2018 from CLSI [7] and from 2017 from EUCAST [8], [9] were assessed for significant differences. We also examined the impact of discrepancies in CLSI and EUCAST zone diameter breakpoints on the interpretation of antimicrobial susceptibility of frequently isolated Gram-negative organisms at one of our sites—the Mahosot Microbiology Laboratory, Vientiane, Laos—in 2017, as reported in this issue of CMI [10]. Although a formal cost analysis was not performed, major additional expenses or savings associated with adopting EUCAST were estimated where possible. Finally, in order to place our findings in a global context, a literature search was performed for articles reporting on EUCAST implementation or differences between EUCAST and CLSI (see web-only Supplementary Fig. S1 and Supplementary Table S1).

Recommendations for inoculum preparation, agar inoculation, disk application and zone diameter reading are similar. Key practical differences are summarized in Table 1. EUCAST recommends an incubation temperature of 35 ± 1°C for 16–20 h for AST of all organisms except *Campylobacter* spp. and when testing glycopeptides against *Enterococcus* spp., which require 24 h in both methodologies. CLSI incubation durations are more variable, and the recommended temperature is 35 ± 2°C. Both systems use Mueller–Hinton agar (MHA) for AST of Enterobacteriaceae, *Pseudomonas* spp., *Staphylococcus* spp., *Acinetobacter* spp., and *Enterococcus* spp., but CLSI uses sheep blood for MHA supplementation whereas EUCAST uses horse blood. Discordant disk contents are used for 13 antimicrobial agents.

**Table 1:** Key differences between the Clinical and Laboratory Standards Institute (CLSI) and the European Committee for Antimicrobial Susceptibility Testing (EUCAST) disk diffusion methodologies

Methodological discrepancy		CLSI	EUCAST
Incubation temperature		35 ± 2°C	35 ± 1°C
Duration of incubation		16–18 h for Enterobacteriaceae, *Staphylococcus* spp.^a^ and *Pseudomonas aeruginosa*; 20–24 h for most other organisms	16–20 h
Media		MHA supplemented with 5% sheep blood for *Streptococcus* spp., *Campylobacter jejuni/coli, Pasteurella* spp. *and Neisseria meningitidis*; Haemophilus Test Medium for *Haemophilus* spp.; GC agar base with 1% defined growth supplement for *Neisseria gonorrhoeae*	MH-F agar for *Streptococcus* spp., *Campylobacter jejuni/coli, Pasteurella* spp.*, H. influenzae*, *Moraxella catarrhalis*, *Listeria monocytogenes*, *Kingella kingae, Aerococcus* spp. and *Corynebacterium* spp.
Antimicrobial disk contents (μg unless otherwise stated)	Amoxicillin–clavulanic acid	20–10	20–10 and 2–1^b^
	Ampicillin	10	10 and 2^c^
	Cefotaxime	30	5
	Ceftaroline	30	5
	Ceftazidime	30	10
	Ceftazidime–avibactam	30–20	10–4
	Gentamicin^d^	120	30
	Linezolid	30	10
	Nitrofurantoin	300	100
	Penicillin G	10 units	1 unit
	Piperacillin	100	30
	Piperacillin–tazobactam	100–10	30–6
	Vancomycin	30	5
Zone diameter breakpoints		None for *Aerococcus* spp., *Kingella kingae, Listeria monocytogenes, Corynebacterium* spp. Rarely aligned with EUCAST	None for *N. gonorrhoeae, N. meningitidis, Burkholderia cepacia, Vibrio* spp. Fewer intermediate categories
ATCC strains for routine disk diffusion quality control		*Staphylococcus aureus* ATCC 25923, *H. influenzae* ATCC 49247	*S. aureus* ATCC 29213, *H. influenzae* ATCC 49766

MHA, Mueller–Hinton agar; MH-F agar, MHA supplemented with 5% horse blood and 20 mg/L β-NAD.

a Except when testing cefoxitin against coagulase-negative staphylococci which requires 24 h.

b For testing *H. influenzae*, *Pasteurella multocida*, *Moraxella catarrhalis*, and *Listeria monocytogenes*_*.*_

c For testing *H. influenzae*, viridans group streptococci, *Staphylococcus saprophyticus* and *Enterococcus* spp.

d For testing high-level resistance in *Enterococcus* spp.

Comparison of published zone diameter breakpoints revealed important differences. CLSI provides zone diameter breakpoints for *Neisseria gonorrhoeae* and *N. meningitidis*, whereas EUCAST has so far deemed disk diffusion unreliable for these organisms and provides only MIC criteria. Within EUCAST breakpoints for Enterobacteriaceae there are no zone diameter criteria for azithromycin–*Salmonella* Typhi, and the nitrofurantoin breakpoint applies only to urinary *Escherichia coli* with the justification that other members of the Enterobacteriaceae are more likely to cause complicated or upper urinary tract infections for which nitrofurantoin is inappropriate. Breakpoints are rarely aligned between the two systems, and the proportion containing an intermediate category is markedly higher in CLSI guidelines. Using the Enterobacteriaceae as an example, 64/69 (93%) of CLSI breakpoints included an intermediate category compared to 24/48 (50%) with EUCAST, and EUCAST resistance cut-offs were higher in 26/33 directly comparable zone diameter breakpoints. Our susceptibility interpretation comparison [10] demonstrated that these discrepancies would have significantly increased reported co-amoxiclav and ciprofloxacin resistance rates amongst clinical isolates of *E. coli* and *Klebsiella pneumoniae* had EUCAST 2018 breakpoints been used. It must be noted that from 1st January 2019, EUCAST changed its definition of the intermediate category to ‘Susceptible, increased exposure’ [11] for when there is a high likelihood of therapeutic success because exposure to the agent is increased by adjusting the dosing regimen or by its concentration at the site of infection. As well as providing unequivocal interpretation of this category, this aims to extend the range of therapeutic options in an era of increasing AMR. The previous definition was more aligned with that of the CLSI as an area of uncertain therapeutic efficacy that included a buffer zone to allow for uncontrolled technical variation. Reporting of results in laboratories adopting EUCAST breakpoints will therefore need to reflect the new definition, requiring additional engagement with laboratory users.

Routine quality control recommendations for disk-diffusion AST are broadly similar. Both organizations advocate daily quality control testing of antimicrobial agents that form part of routine panels, although CLSI allows weekly testing if results on a sufficient number of consecutive days are within range. The same American Type Culture Collection (ATCC) strains of *E. coli*, *Pseudomonas aeruginosa*, *Enterococcus faecalis*, *Streptococcus pneumoniae*, and *Campylobacter jejuni* are employed by both systems, but different ATCC strains of *Staphylococcus aureus* and *Haemophilus influenzae* are used.

Two significant cost implications of switching to EUCAST were identified. Losing the requirement to purchase CLSI documents annually could save approximately US$342–504 per year. However, lack of EUCAST zone diameter breakpoints would require the introduction of a commercial MIC method—e.g. Etest (bioMérieux, France)—for the AST of *N. gonorrhoeae*. At the Mahosot Microbiology Laboratory, where 82 *N. gonorrhoeae* isolates underwent disk-diffusion AST in 2017 against five antimicrobial agents, the added cost of using Etests for these same agents would have been approximately US$1200.

Difficulties in acquiring quality-assured horse blood in Southeast Asia pose a significant barrier to adopting EUCAST media requirements for AST of fastidious organisms. As is common practice in resource-constrained settings, our laboratories in Laos and Cambodia prepare agar plates on site, relying on local sources of goat and sheep blood respectively. It is not currently feasible to obtain horse blood or to import pre-poured plates in these countries, and only MHA supplemented with 5% sheep blood is available from our commercial supplier in Thailand (Clinical Diagnostics Ltd, Bangkok, Thailand). Goat blood is used at Mahosot Microbiology Laboratory as it has been shown to be a reasonable substitute for sheep blood [12], which is unavailable in Laos. Another common source of blood for media supplementation in low-income countries is expired banked human blood, which is suboptimal for the isolation and AST of fastidious organisms [13]. As many laboratories purporting to follow CLSI guidelines will be using more accessible alternatives to sheep blood, challenges in adhering to media guidelines in resource-constrained settings are not unique to EUCAST. Practical solutions are required, and to this end EUCAST is exploring the use of freeze-dried horse blood as a supplement for locally prepared media (G. Kahlmeter, personal communication 2017).

Our literature search (see web-only Supplementary Fig. S1 and Supplementary Table S1) identified 101 articles in PubMed reporting on EUCAST implementation (*n* = 8), specific methodological discrepancies between CLSI and EUCAST (*n* = 20), and comparisons of CLSI and EUCAST breakpoints for susceptibility interpretation (*n* = 73). The susceptibility comparisons generally reported higher resistance rates with EUCAST breakpoints, which our own findings supported [10], and all articles about EUCAST implementation were from Europe, focusing mainly on the history and structure of EUCAST, organizational differences with CLSI, and EUCAST breakpoint-setting and disk-diffusion methodologies. One recent article [14] proposes a roadmap for transitioning from CLSI to EUCAST in Spanish laboratories, providing useful general recommendations and highlighting differences between CLSI and EUCAST such as antimicrobial disk contents and media. However, its broader utility may be limited as it is specifically orientated towards Spanish laboratories and is available only in Spanish. Although we found no articles discussing EUCAST implementation in low- and middle-income countries, we anticipate difficulties with meeting EUCAST standards for blood-supplemented media and AST of *N. gonorrhoeae* to be broadly applicable across resource-constrained settings.

Despite compelling reasons to adopt EUCAST AST guidelines, following discussion at network meetings in 2017 and early in 2018 the consensus decision amongst MORU clinical microbiologists was not to do so at the time. The main reasons behind this were the absence of a national push to change and the need to keep our data comparable with those of other laboratories in the countries in which we work, highlighting another potential barrier to EUCAST implementation outside of Europe. While EUCAST implementation in Europe was driven and coordinated by well-established national committees, it may be challenging for individual laboratories in other regions to switch from CLSI to EUCAST without a unified drive to change at the national level. During the preparation of this manuscript there has been renewed interest in adopting EUCAST methodology in Laos, and a switch from CLSI to EUCAST is planned at Mahosot Microbiology Laboratory and nationally during 2019, albeit with continued use of local sources of blood for media supplementation and retention of CLSI disk diffusion standards for AST of *N. gonorrhoeae*. Our laboratories in Thailand and Cambodia will continue with CLSI.

In conclusion, there are important practical differences between CLSI and EUCAST disk-diffusion AST guidelines, and whilst we agree with other authors advocating EUCAST as the preferred AST methodology in low- and middle-income countries [15], challenges involved in switching to EUCAST from CLSI should not be underestimated in these settings. Furthermore, discrepancies in clinical breakpoints will alter institutional antibiograms following a switch between the two methodologies, and will hamper broader AMR surveillance initiatives such as GLASS. This report highlights the need for a globally harmonized AST system that is practical and freely available, and we hope it will be a useful guide for laboratories considering switching between CLSI and EUCAST.

## Transparency declaration

No payment or services from a third party for any aspect of the submitted work were received by any of the authors or their institutions. None of the authors have financial relationships with entities in the biomedical arena that could be perceived to influence what is written in this manuscript. None of the authors have planned, pending or issued patents broadly relevant to the work, and no authors have other relationships/conditions/circumstances that present a potential conflict of interest. The MORU Tropical Health Network is core funded by Wellcome (grant number 106698/Z/14/Z). The funding body had no role in the design of the study, the collection, analysis, and interpretation of the data, or the writing of the manuscript.

## Author contributions

The work was conceived by all authors at a regional MORU microbiology meeting. TC performed the comparisons of methodology, susceptibility interpretation of clinical isolates, and MIC breakpoints for GLASS priority pathogen–antimicrobial combinations. TC prepared the first draft of the manuscript, the content of which was reviewed and discussed by all authors on several occasions. All authors read and approved the final manuscript.
