# Supplementary material for: Time to switch from CLSI to EUCAST? A Southeast Asian perspective
Source: Clin Microbiol Infect. 2019 Jul;25(7):782–5. doi: 10.1016/j.cmi.2019.03.016 (PMC6587905; doi:10.1016/j.cmi.2019.03.016)
Supplement: Multimedia component 1 [file mmc1.pdf]

Supplementary Material

Supplementary Figure S1. Literature search flow diagram

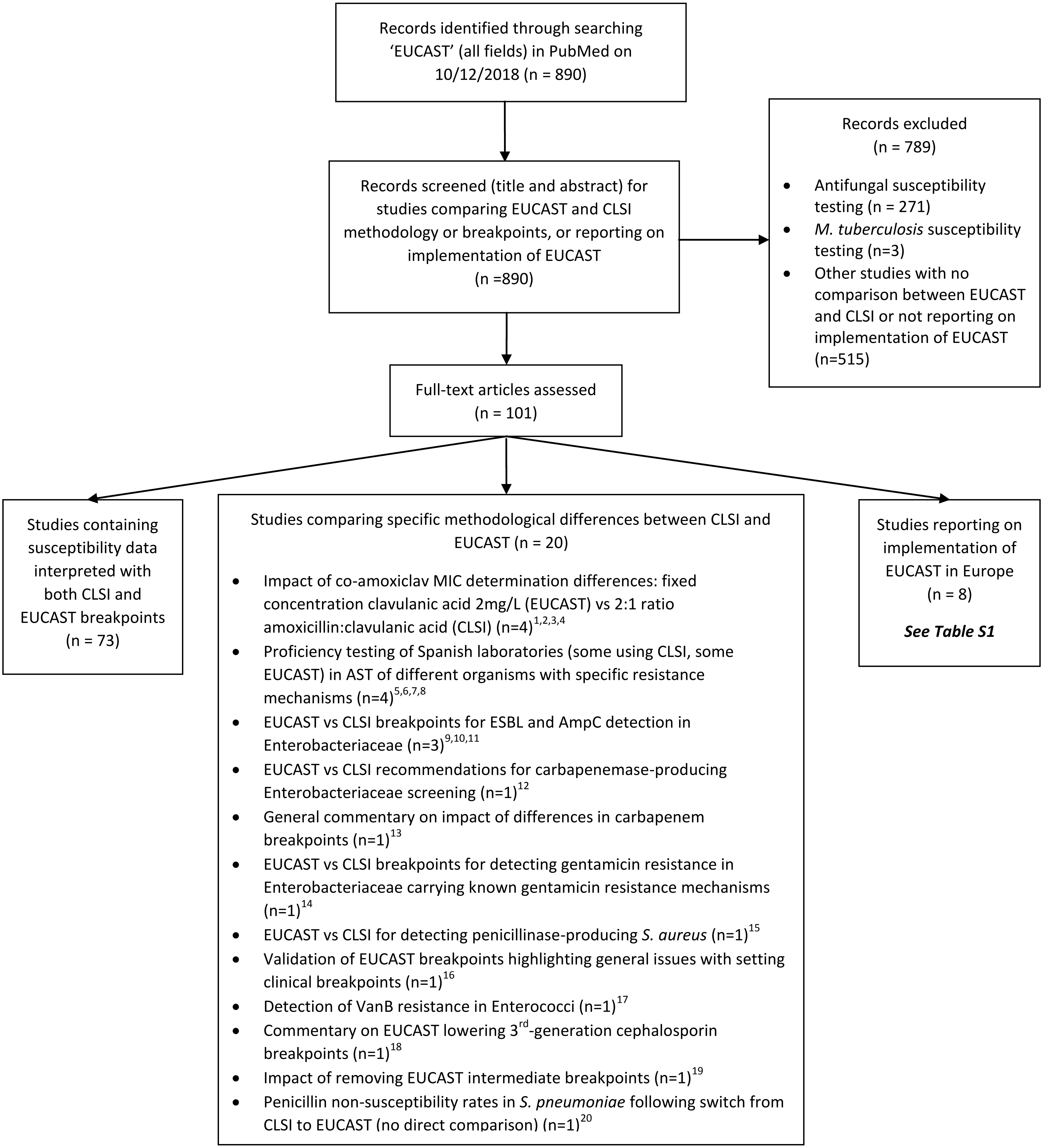

Supplementary Figure S1 References

1. Delgado-Valverde M, Valiente-Mendez A, Torres E, Almirante, B, Gómez-Zorrilla S, Borrell N et al. MIC of amoxicillin/clavulanate according to CLSI and EUCAST: discrepancies and clinical impact in patients with bloodstream infections due to Enterobacteriaceae. J Antimicrob Chemother. 2017;72(5):1478-1487.
2. Díez-Aguilar M., Morosini M-I., López-Cerero L., Pascual Á., Calvo J., Martínez-Martínez L. et al. Performance of EUCAST and CLSI approaches for co-amoxiclav susceptibility testing conditions for clinical categorization of a collection of *Escherichia coli* isolates with characterized resistance phenotypes. J Antimicrob Chemother. 2015;70(8):2306–2310.
3. Leverstein-van Hall MA, Waar K, Muilwijk J, Cohen Stuart J, ISIS-AR Study Group. Consequences of switching from a fixed 2:1 ratio of amoxicillin/clavulanate (CLSI) to a fixed concentration of clavulanate (EUCAST) for susceptibility testing of Escherichia coli. J Antimicrob Chemother. 2013;68(11):2636–2640.
4. María DA, María-Isabel M, María-Carmen C, Álvaro P, Jorge C, Luis MM et al. Establishing the validity of different susceptibility testing methods to evaluate the in vitro activity of amoxicillin-clavulanate against Escherichia coli. Diagn Microbiol Infect Dis. 2016;84(4):334-6.
5. Díez-Aguilar M, Conejo MC, Morosini MI, Tormo Palop N, Gimeno C, Cantón R et al. Susceptibility testing and detection of β-lactam resistance mechanisms in Enterobacteriaceae: a multicentre national proficiency study. Int J Antimicrob Agents. 2018;51(4):612-619.
6. Fernández-Cuenca F, Tomás M, Caballero-Moyano FJ, Bou G, Pascual Á; Spanish Society of Clinical Microbiology and Infectious Diseases (SEIMC). Reporting antimicrobial susceptibilities and resistance phenotypes in Acinetobacter spp: a nationwide proficiency study. J Antimicrob Chemother. 2018;73(3):692-697.
7. Juan C, Conejo MC, Tormo N, Gimeno C, Pascual Á, Oliver A. Challenges for accurate susceptibility testing, detection and interpretation of β-lactam resistance phenotypes in Pseudomonas aeruginosa: results from a Spanish multicentre study. J Antimicrob Chemother. 2013;68: 619–630.
8. Rodríguez-Martínez JM, Machuca J, Calvo J, Díaz-de-Alba P, Rodríguez-Mirones C, Gimeno C et al. Challenges to accurate susceptibility testing and interpretation of quinolone resistance in *Enterobacteriaceae*: results of a Spanish multicentre study. J Antimicrob Chemother. 2015;70:2038–2047.
9. Polsfuss S, Bloemberg GV, Giger J, Meyer V, Hombach M. Comparison of European Committee on Antimicrobial Susceptibility Testing (EUCAST) and CLSI screening parameters for the detection of extended-spectrum β-lactamase production in clinical enterobacteriaceae isolates. J Antimicrob Chemother. 2012(67):159–166.
10. Nastro M, Montoto Piazza L, Saposnik E, García S, Barberis C, Vay C et al . Resistance to extended-spectrum cephalosporins in non-inducible AmpC enterobacteria: Evaluation of the new MIC breakpoints. Rev. argent. microbiol. 2012;44(1): 30-35.
11. Morrissey I, Bouchillon SK, Hackel M, Biedenbach DJ, Hawser S, Hoban D, et al. Evaluation of the Clinical and Laboratory Standards Institute phenotypic confirmatory test to detect the presence of extended-spectrum β-lactamases from 4005 *Escherichia coli*, *Klebsiella oxytoca*, *Klebsiella pneumoniae* and *Proteus mirabilis* isolates. J Med Microbiol. 2014;63: 556–561.
12. Fattouh R, Tijet N, McGeer A, Poutanen SM, Melano RG, Patel SN. What Is the Appropriate Meropenem MIC for Screening of Carbapenemase-Producing Enterobacteriaceae in Low-Prevalence Settings? Antimicrob Agents Chemother. 2016;60: 1556–9.

13. Cantón R, Canut A, Morosini MI, Oliver A. Breakpoints for carbapenemase-producing *Enterobacteriaceae*: is the problem solved? Enferm Infecc Microbiol Clin 2014; 32(Suppl 4):33–40.
14. Jakobsen L, Sandvang D, Jensen VF, Seyfarth AM, Frimodt-Moller N, Hammerum AM. Gentamicin susceptibility in Escherichia coli related to the genetic background: problems with breakpoints. Clin Microbiol Infect. 2007;13:830–832.
15. Papanicolas LE, Bell JM, Bastian I. 2014. Performance of phenotypic tests for detection of penicillinase in Staphylococcus aureus isolates from Australia. J Clin Microbiol 52:1136–1138.
16. Hombach M., Courvalin P., Böttger E. C. Validation of antibiotic susceptibility testing guidelines in a routine clinical microbiology laboratory exemplifies general key challenges in setting clinical breakpoints. Antimicrobial Agents Chemother. 2014;58, 3921–3926.
17. Hegstad K., Giske C. G., Haldorsen B., Matuschek E., Schonning K., Leegaard T. M., et al. Performance of the EUCAST disk diffusion method, the CLSI agar screen method, and the Vitek 2 automated antimicrobial susceptibility testing system for detection of clinical isolates of enterococci with low- and medium-level VanB-type vancomycin resistance: a multicenter study. *J. Clin. Microbiol.* 2014;52 1582–1589. 10.1128/JCM.03544-13
18. Kahlmeter G. Breakpoints for intravenously used cephalosporins in Enterobacteriaceae—EUCAST and CLSI breakpoints. Clin Microbiol Infect. 2008;14 Suppl 1: 169–174.
19. Hombach M, Bottger EC, Roos M. The critical influence of the intermediate category on interpretation errors in revised EUCAST and CLSI antimicrobial susceptibility testing guidelines. Clin Microbiol Infect. 2013;19:E59–E71.
20. Goossens MC, Catry B, Verhaegen J. Antimicrobial resistance to benzylpenicillin in invasive pneumococcal disease in Belgium, 2003–2010: the effect of altering clinical breakpoints. Epidemiol Infect. 2013;141:490–495.

Supplementary Table S1. Summary of studies reporting on implementation of EUCAST in Europe.

Author and Year (Pubmed ID)	Focus	Key points
Brown 2010 (19996143)	Commentary on rationale behind and process of harmonizing breakpoints in Europe under EUCAST, implications of adopting EUCAST on reporting of intermediate susceptibility in UK	Most breakpoints similar or identical between BSAC and EUCAST but some examples of where discrepancies have significant impact on susceptibility interpretation eg ertapenem-*Enterobacter* spp., gentamicin-*P. aeruginosa*. Highlights one area of potential confusion being more extensive use of intermediate category in EUCAST guidelines compared to BSAC and the rationale behind this (BSAC regarded intermediate category as of limited value previously but greater confidence in the intermediate category with recent approaches to setting breakpoints in the harmonization process). Highlights need for harmonization of EUCAST with CLSI – several barriers to this discussed including differences in organizational structures and funding, relationships with regulatory authorities.
Brown 2015 (25613780)	Review of uptake of EUCAST guidelines in European countries	Rapid implementation of EUCAST breakpoints in Europe over the period 2009-2013
Brown 2016 (26377864)	Short review of history of EUCAST and transition from BSAC to EUCAST in the UK including rationale for switching	Key benefits of switching from BSAC to EUCAST disk diffusion method are its correlation with MICs, more antimicrobial agent/organism combinations covered by EUCAST, standardization across Europe and recognition of EUCAST by European Medicines Agency (EMA) for setting breakpoints for new agents
Kahlmeter 2014 (24836050)	Review of history of EUCAST and its implementation in European countries, rationale for harmonization of breakpoints, breakpoint-setting processes, EUCAST structure and relationship with regulatory agencies	Highlights that until 2002 globally there were at least 7 different interpretive systems for AST, making comparison of AMR rates difficult. Some examples of differences in breakpoints between these systems highlighted (cefotaxime-*E. coli* and gentamicin-*E. coli*).
Kahlmeter 2015 (26089441)	History of EUCAST and its implementation in European countries, organisation of EUCAST and structural differences with CLSI, summary of breakpoint	Several important structural differences between CLSI and EUCAST highlighted as reasons why merging of the organisations and harmonization of breakpoints has not occurred:<br>1. <u>Funding:</u> EUCAST is funded by national breakpoint committees, the European Centre for Disease Prevention and Control, and the European Society of Clinical

	setting processes	Microbiology and Infectious Diseases, whereas CLSI is supported by member subscription fees and sales of documents<br>2. Relationship with regulatory agencies: the EMA routinely adopts EUCAST breakpoints, while in the United States the Food and Drug Administration determines breakpoints independently of CLSI<br>3. Relationship with industry: Industry representatives sit on the CLSI Subcommittee on AST Standards and can vote on breakpoint decisions, but have no formal positions on EUCAST committees.
Larrosa 2018 (30409509)	'Roadmap' for Spanish laboratories to transition to EUCAST from CLSI	10 general recommendations:<br>1. Anticipate differences in EUCAST breakpoints and rules of interpretation<br>2. Communicate with commercial suppliers about adapting relevant automated or semi-automated system software to EUCAST criteria<br>3. Adopt disk diffusion technique to specific EUCAST recommendations e.g. incubation durations<br>4. Anticipate changes of antibiotic disk contents and media<br>5. Decide interpretive criteria to be used when EUCAST breakpoints not available<br>6. Ensure that appropriate control strains are available<br>7. Make the necessary changes to laboratory documents and information management systems<br>8. Inform laboratory users of switch to EUCAST and potential differences in susceptibility interpretation<br>9. Consider effect of introduction of EUCAST breakpoints (leading to a reduction in susceptibility rates)<br>10. Consult the Spanish National Antibiogram Committee (COESANT) for any transitional issues
Martinez-Martinez 2013 (24269101)	Editorial on harmonization of COESANT with EUCAST – brief history of EUCAST and its adoption in Europe and role of COESANT in implementation of EUCAST in Spain	Organisational and conceptual differences between CLSI and EUCAST highlighted: lack of industry voting in EUCAST breakpoint decisions, all EUCAST breakpoint and technical documents available for free.
Matuschek 2014 (24131428)	Summary of the EUCAST disk diffusion methodology and its development and general guidance points on its implementation in routine microbiology laboratories	Some differences between CLSI and EUCAST methodologies highlighted including differences in disk concentrations, standard incubation times and quality control criteria.

Supplementary Table S1 References

1. Brown D, Macgowan A. Harmonization of antimicrobial susceptibility testing breakpoints in Europe: Implications for reporting intermediate susceptibility. J Antimicrob Chemother. 2010;65:183–5.
2. Brown D, Canton R, Dubreuil L, Gatermann S, Giske C, MacGowan A, et al. Widespread implementation of EUCAST breakpoints for antibacterial susceptibility testing in Europe. Euro Surveill. 2015; 20(2):21008.10.2807/1560-7917.ES2015.20.2.21008
3. Brown DF, Wootton M, Howe RA. Antimicrobial susceptibility testing breakpoints and methods from BSAC to EUCAST. J Antimicrob Chemother. 2016;71(1):3–5.
4. Kahlmeter G. The 2014 Garrod lecture: EUCAST–are we heading towards international agreement? J Antimicrob Chemother. 2015;70:2427–2439.
5. Kahlmeter G. Defining antibiotic resistance-towards international harmonization. Ups J Med Sci. 2014;119(2):78–86.10.3109/03009734.2014.901446
6. Larrosa MN, Benito N, Canton R, Canut A, Cercenado E, Fernandez-Cuenca F et al. From CLSI to EUCAST, a necessary step in Spanish laboratories. Enferm Infecc Microbiol Clin. 2018. https://doi.org/10.1016/j.eimc.2018.09.014
7. Martínez-Martínez L, Pascual A, Cantón R. The Spanish antibiogram committee (COESANT), in step with EUCAST. Enferm Infecc Microbiol Clin. 2013 Dec;31(10):639-40.
8. Matuschek E, Brown DF, Kahlmeter G. Development of the EUCAST disk diffusion antimicrobial susceptibility testing method and its implementation in routine microbiology laboratories. Clinical Microbiology and Infection. 2014;20(4), O255–O266.
